# Supplementary material for: LPF-Defense: 3D adversarial defense based on frequency analysis
Source: PLoS One. 2023 Feb 6;18(2):e0271388. doi: 10.1371/journal.pone.0271388 (PMC9901796; doi:10.1371/journal.pone.0271388)
Supplement: S1 Table — compares the various defense methods. The column entitled “Type” specifies the general technique that is used in each defense method. In the column entitled “Method”, a brief description of the defense method is introduced to give a better perspective. Finally, the column “strength” indicates each method’s level of success on three main attack mechanisms. Note that the information in this column is general, and the reader might refer to Tables 1–5 for a detailed comparison. Note that the defense methods of type modified input mainly focus on outlier removing and generating a uniform distribution of the points in the point cloud. On the other hand, the defense methods of type modified training train the model with the original data as well as the attack data (which contains outliers). Finally, the proposed methods take advantage of both types by training with the original data while removing the high frequency information. This covers the disadvantage of the modified training idea due to removal of outliers considered as the high frequency components. (PDF) [file pone.0271388.s001.pdf]

**Defenses**	**Type**	**Method**	**strength**		
			Point add	Point shift	Point drop
SRS [25]	input	Outlier removal is done by randomly dropping points.	***	**	*
SOR [33]	input	Outlier removal is done based on the point distance to its k-nearest neighbors.	***	**	*
DUP-Net [33]	input	Outlier removal is done by SOR<br>Uniform distribution of points is achieved using the upsampler network.	***	***	**
If-Defense [34]	input	Outlier removal is done based on limiting the point perturbation with new loss definition + SOR<br>Uniform distribution of points is achieved by imposing penalty on close points using new loss definition.	****	****	***
Adv Training (Shift-L2) [22]	Training	Trains the model with original data and Shift-L2 attacked data.<br>training is done with add adversarial outliers	**	*	***
Adv Training (PAGN) [37]	Training	Trains the model with original data and adaptive attacked data.<br>training is done with add adversarial outliers with outlier-inject-module	**	***	**
LPF1-Proposed	Training /input	Trains the model with information of Low-Freq data.<br>Training is done by removing high-freq information as outliers.<br>Outlier removing is done by LF+SOR.	****	****	****
LPF2-Proposed	Training /input	Trains the model with original data and the information of Low-Freq data.<br>Training is done by removing high-freq information as outliers.<br>Outlier removing is done by LF+SOR.	****	****	*****
