# Supplementary material for: LPF-Defense: 3D adversarial defense based on frequency analysis
Source: PLoS One. 2023 Feb 6;18(2):e0271388. doi: 10.1371/journal.pone.0271388 (PMC9901796; doi:10.1371/journal.pone.0271388)
Supplement: S2 Table — [F1-scores, precision, and recall results for each defense are shown in the first, second, and third row, as a percentage, respectively]. In order to analyze the performance, a confusion matrix is calculated, and then the accuracy, f1-score, precision, and recall criteria are derived from it. S2 Table displays that the proposed method performs well with all three different metrics in the ShapeNet dataset on the PointNet model. (PDF) [file pone.0271388.s002.pdf]

**Defenses**	**Attacks**						
	Clean	Shift-L2 [14]	Add-CD [14]	Add-HD [14]	Shift-KNN [18]	Drop-100 [27]	Drop-200 [27]
SRS [25]	**87.7%**	74.5%	68.6%	65.2%	84.9%	72.2%	57.8%
	84%	76.2%	72.6%	70.6%	81.9%	73.3%	62.9%
	**96.2%**	75%	67.4%	63.5%	89.4%	72.8%	56.5%
SOR [33]	88.8%	83.3%	85.3%	84%	**86%**	69.5%	60.2%
	**85**%	78.9%	80.8%	79.9%	**83%**	71%	64.8%
	96.3%	92.16%	**95.7%**	91.8%	90.4%	69.6%	58.6%
DUP-Net [33]	77.6%	81.5%	83.7%	85.3%	84.2%	69.7%	60.8%
	76.1%	78.16%	81.1%	81.8%	82%	70.7%	64.8%
	85.4%	91.6%	91.7%	**93.7**%	89.5%	70.7%	59.6%
If-Defense [34]	85.6%	85.4%	84.2%	84.3%	82.05%	75.5%	64.8%
	82.2%	81.7%	82%	81.5%	77.8%	75.9%	68.2%
	92.7%	**93.8**%	92.2%	93.4%	**93.3**%	76.6%	63.1%
LPF1-Proposed	74.2%	75.3%	77.4%	77.6%	74.9%	76.9%	66%
	72.9%	72.8%	74.3%	74.8%	71.8%	77%	70.9%
	77.9%	84.7%	90%	87.1%	82.1%	85.8%	69%
LPF2-Proposed	86.6%	**88.6**%	**88.5**%	**89.7**%	83.6%	**84.8%**	**78.1%**
	82.9%	**86.3%**	**85.7%**	**87.6%**	82.9%	**82.7%**	**79.4**%
	93.4%	91.5%	93.7%	92.5%	88.01%	**88.7%**	**77.5%**

References

1. Voulodimos A, Doulamis N, Doulamis A, Protopapadakis E. Deep learning for computer vision: A brief review. Computational intelligence and neuroscience. 2018;2018.
2. Fernandes D, Silva A, Névoa R, Simões C, Gonzalez D, Guevara M, et al. Point-cloud based 3D object detection and classification methods for self-driving applications: A survey and taxonomy. Information Fusion. 2021;68:161–191.
3. Miotto R, Wang F, Wang S, Jiang X, Dudley JT. Deep learning for healthcare: review, opportunities and challenges. Briefings in bioinformatics. 2018;19(6):1236–1246.
4. Qi CR, Su H, Mo K, Guibas LJ. Pointnet: Deep learning on point sets for 3d classification and segmentation. In: Proceedings of the IEEE conference on computer vision and pattern recognition; 2017. p. 652–660.
5. Qi CR, Yi L, Su H, Guibas LJ. PointNet++: Deep Hierarchical Feature Learning on Point Sets in a Metric Space; 2017.
6. Phan AV, Le Nguyen M, Nguyen YLH, Bui LT. Dgcnn: A convolutional neural network over large-scale labeled graphs. Neural Networks. 2018;108:533–543.
7. Moosavi-Dezfooli SM, Fawzi A, Frossard P. Deepfool: a simple and accurate method to fool deep neural networks. In: Proceedings of the IEEE conference on computer vision and pattern recognition; 2016. p. 2574–2582.
8. Naderi H, Goli L, Kasaei S. Generating Unrestricted Adversarial Examples via Three Parameters. Multimedia Tools and Applications. 2022;-(-):–.
9. Carlini N, Wagner D. Towards evaluating the robustness of neural networks. In: 2017 ieee symposium on security and privacy (sp). IEEE; 2017. p. 39–57.
10. Goodfellow IJ, Shlens J, Szegedy C. Explaining and Harnessing Adversarial Examples; 2015.
11. An Y, Li Z, Shao C. Feature extraction from 3D point cloud data based on discrete curves. Mathematical Problems in Engineering. 2013;2013.
12. Naderi H, Goli L, Kasaei S. Scale Equivariant CNNs with Scale Steerable Filters. In: 2020 International Conference on Machine Vision and Image Processing (MVIP). IEEE; 2020. p. 1–5.
13. Madry A, Makelov A, Schmidt L, Tsipras D, Vladu A. Towards Deep Learning Models Resistant to Adversarial Attacks; 2019.
14. Xiang C, Qi CR, Li B. Generating 3d adversarial point clouds. In: Proceedings of the IEEE/CVF Conference on Computer Vision and Pattern Recognition; 2019. p. 9136–9144.
15. Hamdi A, Rojas S, Thabet A, Ghanem B. Advpc: Transferable adversarial perturbations on 3d point clouds. In: European Conference on Computer Vision. Springer; 2020. p. 241–257.
16. Lee K, Chen Z, Yan X, Urtasun R, Yumer E. Shapeadv: Generating shape-aware adversarial 3d point clouds. arXiv preprint arXiv:200511626. 2020;.

17. Zhou H, Chen D, Liao J, Chen K, Dong X, Liu K, et al. Lg-gan: Label guided adversarial network for flexible targeted attack of point cloud based deep networks. In: Proceedings of the IEEE/CVF Conference on Computer Vision and Pattern Recognition; 2020. p. 10356–10365.

18. Tsai T, Yang K, Ho TY, Jin Y. Robust adversarial objects against deep learning models. In: Proceedings of the AAAI Conference on Artificial Intelligence. vol. 34; 2020. p. 954–962.

19. Wen Y, Lin J, Chen K, Chen CP, Jia K. Geometry-aware generation of adversarial point clouds. IEEE Transactions on Pattern Analysis and Machine Intelligence. 2020;.

20. Hu Q, Liu D, Hu W. Exploring the Devil in Graph Spectral Domain for 3D Point Cloud Attacks. arXiv preprint arXiv:220207261. 2022;.

21. Li K, Zhang Z, Zhong C, Wang G. Robust Structured Declarative Classifiers for 3D Point Clouds: Defending Adversarial Attacks with Implicit Gradients. In: 2022 IEEE/CVF Conference on Computer Vision and Pattern Recognition (CVPR). IEEE; 2022. p. 15294–15304.

22. Liu D, Yu R, Su H. Extending adversarial attacks and defenses to deep 3d point cloud classifiers. In: 2019 IEEE International Conference on Image Processing (ICIP). IEEE; 2019. p. 2279–2283.

23. Arya A, Naderi H, Kasaei S. Adversarial Attack by Limited Point Cloud Surface Modifications. arXiv preprint arXiv:211003745. 2021;.

24. Liu D, Yu R, Su H. Adversarial shape perturbations on 3D point clouds. In: European Conference on Computer Vision. Springer; 2020. p. 88–104.

25. Yang J, Zhang Q, Fang R, Ni B, Liu J, Tian Q. Adversarial Attack and Defense on Point Sets; 2021.

26. Kim J, Hua BS, Nguyen T, Yeung SK. Minimal adversarial examples for deep learning on 3d point clouds. In: Proceedings of the IEEE/CVF International Conference on Computer Vision; 2021. p. 7797–7806.

27. Zheng T, Chen C, Yuan J, Li B, Ren K. Pointcloud saliency maps. In: Proceedings of the IEEE/CVF International Conference on Computer Vision; 2019. p. 1598–1606.

28. Ma C, Meng W, Wu B, Xu S, Zhang X. Efficient joint gradient based attack against sor defense for 3d point cloud classification. In: Proceedings of the 28th ACM International Conference on Multimedia; 2020. p. 1819–1827.

29. Liu D, Hu W. Imperceptible Transfer Attack and Defense on 3D Point Cloud Classification. arXiv preprint arXiv:211110990. 2021;.

30. Wicker M, Kwiatkowska M. Robustness of 3d deep learning in an adversarial setting. In: Proceedings of the IEEE/CVF Conference on Computer Vision and Pattern Recognition; 2019. p. 11767–11775.

31. Liu D, Yu R, Su H. Adversarial point perturbations on 3d objects. arXiv e-prints. 2019; p. arXiv–1908.

32. Dai X, Li Y, Dai H, Xiao B. Generating Unrestricted 3D Adversarial Point Clouds. arXiv preprint arXiv:211108973. 2021;.

33. Zhou H, Chen K, Zhang W, Fang H, Zhou W, Yu N. Dup-net: Denoiser and upsampler network for 3d adversarial point clouds defense. In: Proceedings of the IEEE/CVF International Conference on Computer Vision; 2019. p. 1961–1970.

34. Wu Z, Duan Y, Wang H, Fan Q, Guibas LJ. If-defense: 3d adversarial point cloud defense via implicit function based restoration. arXiv preprint arXiv:201005272. 2020;.

35. Liu H, Jia J, Gong NZ. PointGuard: Provably Robust 3D Point Cloud Classification. In: Proceedings of the IEEE/CVF Conference on Computer Vision and Pattern Recognition; 2021. p. 6186–6195.

36. Dong X, Chen D, Zhou H, Hua G, Zhang W, Yu N. Self-Robust 3D Point Recognition via Gather-Vector Guidance. In: 2020 IEEE/CVF Conference on Computer Vision and Pattern Recognition (CVPR). IEEE; 2020. p. 11513–11521.

37. Liang Q, Li Q, Nie W, Liu AA. PAGN: perturbation adaption generation network for point cloud adversarial defense. Multimedia Systems. 2022; p. 1–9.

38. Sun J, Koenig K, Cao Y, Chen QA, Mao Z. On the adversarial robustness of 3d point cloud classification. 2020;.

39. Sun J, Cao Y, Choy C, Yu Z, Xiao C, Anandkumar A, et al. Improving adversarial robustness in 3D point cloud classification via self-supervisions. In: International Conference on Machine Learning Workshop (ICMLW). vol. 1; 2021.

40. Ilyas A, Santurkar S, Tsipras D, Engstrom L, Tran B, Madry A. Adversarial examples are not bugs, they are features. arXiv preprint arXiv:190502175. 2019;.

41. Wang Z, Yang Y, Shrivastava A, Rawal V, Ding Z. Towards frequency-based explanation for robust cnn. arXiv preprint arXiv:200503141. 2020;.

42. Yin D, Lopes RG, Shlens J, Cubuk ED, Gilmer J. A fourier perspective on model robustness in computer vision. arXiv preprint arXiv:190608988. 2019;.

43. Ortiz-Jimenez G, Modas A, Moosavi-Dezfooli SM, Frossard P. Hold me tight! Influence of discriminative features on deep network boundaries. arXiv preprint arXiv:200206349. 2020;.

44. Guo C, Frank JS, Weinberger KQ. Low frequency adversarial perturbation. arXiv preprint arXiv:180908758. 2018;.

45. Sharma Y, Ding GW, Brubaker M. On the effectiveness of low frequency perturbations. arXiv preprint arXiv:190300073. 2019;.

46. Duan R, Chen Y, Niu D, Yang Y, Qin A, He Y. AdvDrop: Adversarial Attack to DNNs by Dropping Information. In: Proceedings of the IEEE/CVF International Conference on Computer Vision; 2021. p. 7506–7515.

47. Lv B, Yang P, Wang Z, Zhu Z. A frequency domain analysis of gradient-based adversarial examples. 2020;.

48. Song Z, Deng Z. An Adversarial Examples Defense Method Based on Image Low-Frequency Information. In: International Conference on Artificial Intelligence and Security. Springer; 2021. p. 204–213.

49. Wang H, Wu X, Huang Z, Xing EP. High-frequency component helps explain the generalization of convolutional neural networks. In: Proceedings of the IEEE/CVF Conference on Computer Vision and Pattern Recognition; 2020. p. 8684–8694.

50. Han S, Lin C, Shen C, Wang Q. Rethinking Adversarial Examples Exploiting Frequency-Based Analysis. In: International Conference on Information and Communications Security. Springer; 2021. p. 73–89.

51. Liu B, Zhang J, Chen L, Zhu J. Boosting 3D Adversarial Attacks with Attacking On Frequency. arXiv preprint arXiv:220110937. 2022;.

52. R Schmitt PF, Aachen. A 3D-Fourier-Descriptor Approach to Compress and Classify 3D Imaging Data. SENSOR+TEST Conferences 2009. 2009;30:133–138.

53. Huang R, Xu Y, Yao W, Hoegner L, Stilla U. Robust global registration of point clouds by closed-form solution in the frequency domain. ISPRS Journal of Photogrammetry and Remote Sensing. 2021;171:310–329.

54. Huang R, Ye Z, Yao W, et al. RIDF: a robust rotation-invariant descriptor for 3D point cloud registration in the frequency domain. ISPRS Annals of the Photogrammetry, Remote Sensing and Spatial Information Sciences. 2020;.

55. Poulenard A, Rakotosaona MJ, Ponty Y, Ovsjanikov M. Effective rotation-invariant point cnn with spherical harmonics kernels. In: 2019 International Conference on 3D Vision (3DV). IEEE; 2019. p. 47–56.

56. Zhang S, Cui S, Ding Z. Hypergraph spectral analysis and processing in 3D point cloud. IEEE Transactions on Image Processing. 2020;30:1193–1206.

57. Cohen TS, Geiger M, Köhler J, Welling M. Spherical cnns. arXiv preprint arXiv:180110130. 2018;.

58. Ramasinghe S, Khan S, Barnes N, Gould S. Spectral-gans for high-resolution 3d point-cloud generation. In: 2020 IEEE/RSJ International Conference on Intelligent Robots and Systems (IROS). IEEE; 2020. p. 8169–8176.

59. Shen W, Jia Y, Wu Y. 3D shape reconstruction from images in the frequency domain. In: Proceedings of the IEEE/CVF Conference on Computer Vision and Pattern Recognition; 2019. p. 4471–4479.

60. Vranic D, Saupe D. 3D shape descriptor based on 3D Fourier transform. In: EURASIP; 2001. p. 271–274.

61. Tramer F, Carlini N, Brendel W, Madry A. On adaptive attacks to adversarial example defenses. Advances in Neural Information Processing Systems. 2020;33:1633–1645.

62. Dinesh C, Cheung G, Bajić IV. Point cloud denoising via feature graph laplacian regularization. IEEE Transactions on Image Processing. 2020;29:4143–4158.

63. Wieczorek MA, Meschede M. SHTools: Tools for working with spherical harmonics. Geochemistry, Geophysics, Geosystems. 2018;19(8):2574–2592.

64. Wu Z, Song S, Khosla A, Yu F, Zhang L, Tang X, et al. 3d shapenets: A deep representation for volumetric shapes. In: Proceedings of the IEEE conference on computer vision and pattern recognition; 2015. p. 1912–1920.

65. Uy MA, Pham QH, Hua BS, Nguyen T, Yeung SK. Revisiting point cloud classification: A new benchmark dataset and classification model on real-world data. In: Proceedings of the IEEE/CVF international conference on computer vision; 2019. p. 1588–1597.

66. Chang AX, Funkhouser T, Guibas L, Hanrahan P, Huang Q, Li Z, et al. ShapeNet: An Information-Rich 3D Model Repository. Stanford University — Princeton University — Toyota Technological Institute at Chicago; 2015. arXiv:1512.03012 [cs.GR].
