# Supplementary material for: LPF-Defense: 3D adversarial defense based on frequency analysis
Source: PLoS One. 2023 Feb 6;18(2):e0271388. doi: 10.1371/journal.pone.0271388 (PMC9901796; doi:10.1371/journal.pone.0271388)
Supplement: S3 Table — Time column lists the average inference time required to compute one point cloud. S3 Table compares the computational cost for the preprocessing step in different defense methods. It shows the average inference time of one adversarial point cloud (shift-l2) for several defense methods. While the low pass filtering method proposed in this paper is computationally heavier than SOR, SRS, and DUP-Net defense methods, it beats the state-of-the-art IF-Defense. The experiment is conducted on a Google Colab environment with a NVIDIA Tesla T4 GPU. (PDF) [file pone.0271388.s003.pdf]

**Defenses**	**Time (millisecond)**
SRS [25]	0.1ms
SOR [33]	2.6ms
DUP-Net [33]	13.66
If-Defense [34]	164ms
LPF-Proposed	73.86ms

References

1. Voulodimos A, Doulamis N, Doulamis A, Protopapadakis E. Deep learning for computer vision: A brief review. Computational intelligence and neuroscience. 2018;2018.

2. Fernandes D, Silva A, Névoa R, Simões C, Gonzalez D, Guevara M, et al. Point-cloud based 3D object detection and classification methods for self-driving applications: A survey and taxonomy. Information Fusion. 2021;68:161–191.

3. Miotto R, Wang F, Wang S, Jiang X, Dudley JT. Deep learning for healthcare: review, opportunities and challenges. Briefings in bioinformatics. 2018;19(6):1236–1246.

4. Qi CR, Su H, Mo K, Guibas LJ. Pointnet: Deep learning on point sets for 3d classification and segmentation. In: Proceedings of the IEEE conference on computer vision and pattern recognition; 2017. p. 652–660.

5. Qi CR, Yi L, Su H, Guibas LJ. PointNet++: Deep Hierarchical Feature Learning on Point Sets in a Metric Space; 2017.

6. Phan AV, Le Nguyen M, Nguyen YLH, Bui LT. Dgcnn: A convolutional neural network over large-scale labeled graphs. Neural Networks. 2018;108:533–543.

7. Moosavi-Dezfooli SM, Fawzi A, Frossard P. Deepfool: a simple and accurate method to fool deep neural networks. In: Proceedings of the IEEE conference on computer vision and pattern recognition; 2016. p. 2574–2582.

8. Naderi H, Goli L, Kasaei S. Generating Unrestricted Adversarial Examples via Three Parameters. Multimedia Tools and Applications. 2022;-(-):–.

9. Carlini N, Wagner D. Towards evaluating the robustness of neural networks. In: 2017 ieee symposium on security and privacy (sp). IEEE; 2017. p. 39–57.

10. Goodfellow IJ, Shlens J, Szegedy C. Explaining and Harnessing Adversarial Examples; 2015.

11. An Y, Li Z, Shao C. Feature extraction from 3D point cloud data based on discrete curves. Mathematical Problems in Engineering. 2013;2013.

12. Naderi H, Goli L, Kasaei S. Scale Equivariant CNNs with Scale Steerable Filters. In: 2020 International Conference on Machine Vision and Image Processing (MVIP). IEEE; 2020. p. 1–5.

13. Madry A, Makelov A, Schmidt L, Tsipras D, Vladu A. Towards Deep Learning Models Resistant to Adversarial Attacks; 2019.

14. Xiang C, Qi CR, Li B. Generating 3d adversarial point clouds. In: Proceedings of the IEEE/CVF Conference on Computer Vision and Pattern Recognition; 2019. p. 9136–9144.

15. Hamdi A, Rojas S, Thabet A, Ghanem B. Advpc: Transferable adversarial perturbations on 3d point clouds. In: European Conference on Computer Vision. Springer; 2020. p. 241–257.

16. Lee K, Chen Z, Yan X, Urtasun R, Yumer E. Shapeadv: Generating shape-aware adversarial 3d point clouds. arXiv preprint arXiv:200511626. 2020;.

17. Zhou H, Chen D, Liao J, Chen K, Dong X, Liu K, et al. Lg-gan: Label guided adversarial network for flexible targeted attack of point cloud based deep networks. In: Proceedings of the IEEE/CVF Conference on Computer Vision and Pattern Recognition; 2020. p. 10356–10365.

18. Tsai T, Yang K, Ho TY, Jin Y. Robust adversarial objects against deep learning models. In: Proceedings of the AAAI Conference on Artificial Intelligence. vol. 34; 2020. p. 954–962.

19. Wen Y, Lin J, Chen K, Chen CP, Jia K. Geometry-aware generation of adversarial point clouds. IEEE Transactions on Pattern Analysis and Machine Intelligence. 2020;.

20. Hu Q, Liu D, Hu W. Exploring the Devil in Graph Spectral Domain for 3D Point Cloud Attacks. arXiv preprint arXiv:220207261. 2022;.

21. Li K, Zhang Z, Zhong C, Wang G. Robust Structured Declarative Classifiers for 3D Point Clouds: Defending Adversarial Attacks with Implicit Gradients. In: 2022 IEEE/CVF Conference on Computer Vision and Pattern Recognition (CVPR). IEEE; 2022. p. 15294–15304.

22. Liu D, Yu R, Su H. Extending adversarial attacks and defenses to deep 3d point cloud classifiers. In: 2019 IEEE International Conference on Image Processing (ICIP). IEEE; 2019. p. 2279–2283.

23. Arya A, Naderi H, Kasaei S. Adversarial Attack by Limited Point Cloud Surface Modifications. arXiv preprint arXiv:211003745. 2021;.

24. Liu D, Yu R, Su H. Adversarial shape perturbations on 3D point clouds. In: European Conference on Computer Vision. Springer; 2020. p. 88–104.

25. Yang J, Zhang Q, Fang R, Ni B, Liu J, Tian Q. Adversarial Attack and Defense on Point Sets; 2021.

26. Kim J, Hua BS, Nguyen T, Yeung SK. Minimal adversarial examples for deep learning on 3d point clouds. In: Proceedings of the IEEE/CVF International Conference on Computer Vision; 2021. p. 7797–7806.

27. Zheng T, Chen C, Yuan J, Li B, Ren K. Pointcloud saliency maps. In: Proceedings of the IEEE/CVF International Conference on Computer Vision; 2019. p. 1598–1606.

28. Ma C, Meng W, Wu B, Xu S, Zhang X. Efficient joint gradient based attack against sor defense for 3d point cloud classification. In: Proceedings of the 28th ACM International Conference on Multimedia; 2020. p. 1819–1827.

29. Liu D, Hu W. Imperceptible Transfer Attack and Defense on 3D Point Cloud Classification. arXiv preprint arXiv:211110990. 2021;.

30. Wicker M, Kwiatkowska M. Robustness of 3d deep learning in an adversarial setting. In: Proceedings of the IEEE/CVF Conference on Computer Vision and Pattern Recognition; 2019. p. 11767–11775.

31. Liu D, Yu R, Su H. Adversarial point perturbations on 3d objects. arXiv e-prints. 2019; p. arXiv–1908.

32. Dai X, Li Y, Dai H, Xiao B. Generating Unrestricted 3D Adversarial Point Clouds. arXiv preprint arXiv:211108973. 2021;.

33. Zhou H, Chen K, Zhang W, Fang H, Zhou W, Yu N. Dup-net: Denoiser and upsampler network for 3d adversarial point clouds defense. In: Proceedings of the IEEE/CVF International Conference on Computer Vision; 2019. p. 1961–1970.

34. Wu Z, Duan Y, Wang H, Fan Q, Guibas LJ. If-defense: 3d adversarial point cloud defense via implicit function based restoration. arXiv preprint arXiv:201005272. 2020;.

35. Liu H, Jia J, Gong NZ. PointGuard: Provably Robust 3D Point Cloud Classification. In: Proceedings of the IEEE/CVF Conference on Computer Vision and Pattern Recognition; 2021. p. 6186–6195.

36. Dong X, Chen D, Zhou H, Hua G, Zhang W, Yu N. Self-Robust 3D Point Recognition via Gather-Vector Guidance. In: 2020 IEEE/CVF Conference on Computer Vision and Pattern Recognition (CVPR). IEEE; 2020. p. 11513–11521.

37. Liang Q, Li Q, Nie W, Liu AA. PAGN: perturbation adaption generation network for point cloud adversarial defense. Multimedia Systems. 2022; p. 1–9.

38. Sun J, Koenig K, Cao Y, Chen QA, Mao Z. On the adversarial robustness of 3d point cloud classification. 2020;.

39. Sun J, Cao Y, Choy C, Yu Z, Xiao C, Anandkumar A, et al. Improving adversarial robustness in 3D point cloud classification via self-supervisions. In: International Conference on Machine Learning Workshop (ICMLW). vol. 1; 2021.

40. Ilyas A, Santurkar S, Tsipras D, Engstrom L, Tran B, Madry A. Adversarial examples are not bugs, they are features. arXiv preprint arXiv:190502175. 2019;.

41. Wang Z, Yang Y, Shrivastava A, Rawal V, Ding Z. Towards frequency-based explanation for robust cnn. arXiv preprint arXiv:200503141. 2020;.

42. Yin D, Lopes RG, Shlens J, Cubuk ED, Gilmer J. A fourier perspective on model robustness in computer vision. arXiv preprint arXiv:190608988. 2019;.

43. Ortiz-Jimenez G, Modas A, Moosavi-Dezfooli SM, Frossard P. Hold me tight! Influence of discriminative features on deep network boundaries. arXiv preprint arXiv:200206349. 2020;.

44. Guo C, Frank JS, Weinberger KQ. Low frequency adversarial perturbation. arXiv preprint arXiv:180908758. 2018;.

45. Sharma Y, Ding GW, Brubaker M. On the effectiveness of low frequency perturbations. arXiv preprint arXiv:190300073. 2019;.

46. Duan R, Chen Y, Niu D, Yang Y, Qin A, He Y. AdvDrop: Adversarial Attack to DNNs by Dropping Information. In: Proceedings of the IEEE/CVF International Conference on Computer Vision; 2021. p. 7506–7515.

47. Lv B, Yang P, Wang Z, Zhu Z. A frequency domain analysis of gradient-based adversarial examples. 2020;.

48. Song Z, Deng Z. An Adversarial Examples Defense Method Based on Image Low-Frequency Information. In: International Conference on Artificial Intelligence and Security. Springer; 2021. p. 204–213.

49. Wang H, Wu X, Huang Z, Xing EP. High-frequency component helps explain the generalization of convolutional neural networks. In: Proceedings of the IEEE/CVF Conference on Computer Vision and Pattern Recognition; 2020. p. 8684–8694.

50. Han S, Lin C, Shen C, Wang Q. Rethinking Adversarial Examples Exploiting Frequency-Based Analysis. In: International Conference on Information and Communications Security. Springer; 2021. p. 73–89.

51. Liu B, Zhang J, Chen L, Zhu J. Boosting 3D Adversarial Attacks with Attacking On Frequency. arXiv preprint arXiv:220110937. 2022;.

52. R Schmitt PF, Aachen. A 3D-Fourier-Descriptor Approach to Compress and Classify 3D Imaging Data. SENSOR+TEST Conferences 2009. 2009;30:133–138.

53. Huang R, Xu Y, Yao W, Hoegner L, Stilla U. Robust global registration of point clouds by closed-form solution in the frequency domain. ISPRS Journal of Photogrammetry and Remote Sensing. 2021;171:310–329.

54. Huang R, Ye Z, Yao W, et al. RIDF: a robust rotation-invariant descriptor for 3D point cloud registration in the frequency domain. ISPRS Annals of the Photogrammetry, Remote Sensing and Spatial Information Sciences. 2020;.

55. Poulenard A, Rakotosaona MJ, Ponty Y, Ovsjanikov M. Effective rotation-invariant point cnn with spherical harmonics kernels. In: 2019 International Conference on 3D Vision (3DV). IEEE; 2019. p. 47–56.

56. Zhang S, Cui S, Ding Z. Hypergraph spectral analysis and processing in 3D point cloud. IEEE Transactions on Image Processing. 2020;30:1193–1206.

57. Cohen TS, Geiger M, Köhler J, Welling M. Spherical cnns. arXiv preprint arXiv:180110130. 2018;.

58. Ramasinghe S, Khan S, Barnes N, Gould S. Spectral-gans for high-resolution 3d point-cloud generation. In: 2020 IEEE/RSJ International Conference on Intelligent Robots and Systems (IROS). IEEE; 2020. p. 8169–8176.

59. Shen W, Jia Y, Wu Y. 3D shape reconstruction from images in the frequency domain. In: Proceedings of the IEEE/CVF Conference on Computer Vision and Pattern Recognition; 2019. p. 4471–4479.

60. Vranic D, Saupe D. 3D shape descriptor based on 3D Fourier transform. In: EURASIP; 2001. p. 271–274.

61. Tramer F, Carlini N, Brendel W, Madry A. On adaptive attacks to adversarial example defenses. Advances in Neural Information Processing Systems. 2020;33:1633–1645.

62. Dinesh C, Cheung G, Bajić IV. Point cloud denoising via feature graph laplacian regularization. IEEE Transactions on Image Processing. 2020;29:4143–4158.

63. Wieczorek MA, Meschede M. SHTools: Tools for working with spherical harmonics. Geochemistry, Geophysics, Geosystems. 2018;19(8):2574–2592.

64. Wu Z, Song S, Khosla A, Yu F, Zhang L, Tang X, et al. 3d shapenets: A deep representation for volumetric shapes. In: Proceedings of the IEEE conference on computer vision and pattern recognition; 2015. p. 1912–1920.

65. Uy MA, Pham QH, Hua BS, Nguyen T, Yeung SK. Revisiting point cloud classification: A new benchmark dataset and classification model on real-world data. In: Proceedings of the IEEE/CVF international conference on computer vision; 2019. p. 1588–1597.

66. Chang AX, Funkhouser T, Guibas L, Hanrahan P, Huang Q, Li Z, et al. ShapeNet: An Information-Rich 3D Model Repository. Stanford University — Princeton University — Toyota Technological Institute at Chicago; 2015. arXiv:1512.03012 [cs.GR].
